# Alpha-Lipoic Acid Reduces LDL-Particle Number and PCSK9 Concentrations in High-Fat Fed Obese Zucker Rats

**DOI:** 10.1371/journal.pone.0090863

**Published:** 2014-03-04

**Authors:** Bradley Carrier, Shin Wen, Sophia Zigouras, Richard W. Browne, Zhuyun Li, Mulchand S. Patel, David L. Williamson, Todd C. Rideout

**Affiliations:** 1 Departments of Exercise and Nutrition Sciences, School of Public Health and Health Professions, University at Buffalo, Buffalo, New York, United States of America; 2 Biotechnical and Clinical Laboratory Sciences, University at Buffalo, Buffalo, New York, United States of America; 3 Biochemistry, School of Medicine and Biomedical Sciences, University at Buffalo, Buffalo, New York, United States of America; Northeast Ohio Medical University, United States of America

## Abstract

We characterized the hypolipidemic effects of alpha-lipoic acid (LA, R-form) and examined the associated molecular mechanisms in a high fat fed Zucker rat model. Rats (n = 8) were assigned to a high fat (HF) diet or the HF diet with 0.25% LA (HF-LA) for 30 days and pair fed to remove confounding effects associated with the anorectic properties of LA. Compared with the HF controls, the HF-LA group was protected against diet-induced obesity (102.5±3.1 vs. 121.5±3.6,% change BW) and hypercholesterolemia with a reduction in total-C (−21%), non-HDL-C (−25%), LDL-C (−16%), and total LDL particle number (−46%) and an increase in total HDL particles (∼22%). This cholesterol-lowering response was associated with a reduction in plasma PCSK9 concentration (−70%) and an increase in hepatic LDLr receptor protein abundance (2 fold of HF). Compared with the HF-fed animals, livers of LA-supplemented animals were protected against TG accumulation (−46%), likely through multiple mechanisms including: a suppressed lipogenic response (down-regulation of hepatic acetyl-CoA carboxylase and fatty acid synthase expression); enhanced hepatic fat oxidation (increased carnitine palmitoyltransferase Iα expression); and enhanced VLDL export (increased hepatic diacylglycerol acyltransferase and microsomal triglyceride transfer protein expression and elevated plasma VLDL particle number). Study results also support an enhanced fatty acid uptake (2.8 fold increase in total lipase activity) and oxidation (increased CPT1β protein abundance) in muscle tissue in LA-supplemented animals compared with the HF group. In summary, in the absence of a change in caloric intake, LA was effective in protecting against hypercholesterolemia and hepatic fat accumulation under conditions of strong genetic and dietary predisposition toward obesity and dyslipidemia.

## Introduction

Obesity, with a prevalence of over 35% in American adults and 16.9% in children and adolescence, is considered the most critical threat to the health and well being of Americans [1], [2]. Obesity-associated metabolic abnormalities, including insulin resistance, dyslipidemia, and fatty liver contribute substantially to elevated risk of cardiovascular disease (CVD) and diabetes.

The liver plays a central role in regulating whole-body lipid metabolism through lipoprotein assembly and secretion, *de novo* lipogenesis and fat oxidation, and clearance of both diet-derived and *de novo* synthesized fat and cholesterol. Compared with lean, insulin sensitive individuals, hepatic lipid metabolism in obese/insulin resistant states is greatly perturbed with increased *de novo* fatty acid synthesis and an alarming prevalence (>80%) of non-alcoholic fatty liver disease, a condition characterized by multiple hepatic pathologies related to excessive accumulation of hepatic TG [3], [4]. Blood lipid abnormalities in obese, insulin resistant individuals, including increased TG and small dense low-density lipoprotein (LDL) particles, are directly and indirectly linked with the inability of peripheral and hepatic tissues to respond to the normal actions of insulin in regulating fatty acid storage and lipoprotein assembly [5].

Although weight reduction through significant and sustained lifestyle modifications in diet and exercise is effective in improving insulin resistance and the associated metabolic disturbances, there exist limited nutraceutical options specifically recognized to protect against dyslipidemia and hepatic steatosis. However, one promising nutraceutical therapy is α-lipoic acid (LA), a naturally occurring short-chain fatty acid (8 carbons) with two sulphur groups, traditionally recognized as an essential cofactor in mitochondrial respiratory enzymes [6]. LA contains a chiral carbon and therefore exists as both R and S enantiomers. Although the majority of commercial supplements consist of a racemic mix of R, S-LA, previous work suggest that the R form may have increased bioavailability and hence elicit a more pronounced physiological response compared with the S form [6]. Ingestion of LA from dietary sources including muscle, heart, kidney, and liver is low; therefore, the potential health implications of LA have been investigated in human subjects by supplementation studies utilizing wide-ranging doses from 50–1800 mg/d [7]–[9]. As a dietary supplement, LA appears to have broad molecular specificity with an impressive array of metabolic benefits including protection against weight gain [10], diet-induced dyslipidemia [11], arterial lesion formation [12], and insulin resistance [13]. The lipid-modulating effects of LA have been attributed to both direct effects and secondary responses associated with the anorectic properties of the supplement [14]–[17]. Although pre-clinical evidence strongly suggests that LA reduces blood lipids (total-C, LDL-C, and TG), very little is known regarding the effects of LA on lipoprotein distribution and size, even though these endpoints are considered valuable CVD predictive biomarkers. Therefore, the purpose of this study was to evaluate the protective effects of LA on blood lipids and lipoproteins and seek the underlying mechanism by examining gene/protein expression of hepatic regulators of cholesterol metabolism and *de novo* lipogenesis, fatty acid oxidation, and lipoprotein assembly. As crosstalk between genetics and dietary factors influence the development of obesity and associated dyslipidemia, we chose to test the efficacy of LA under the influence of strong genetic and dietary predisposition to obesity and dyslipidemia with the use of high fat-fed Zucker rats, independent of changes in feed or caloric intake induced by LA.

## Results

### Body weight gain, feed and caloric intake

**Figure 1:**
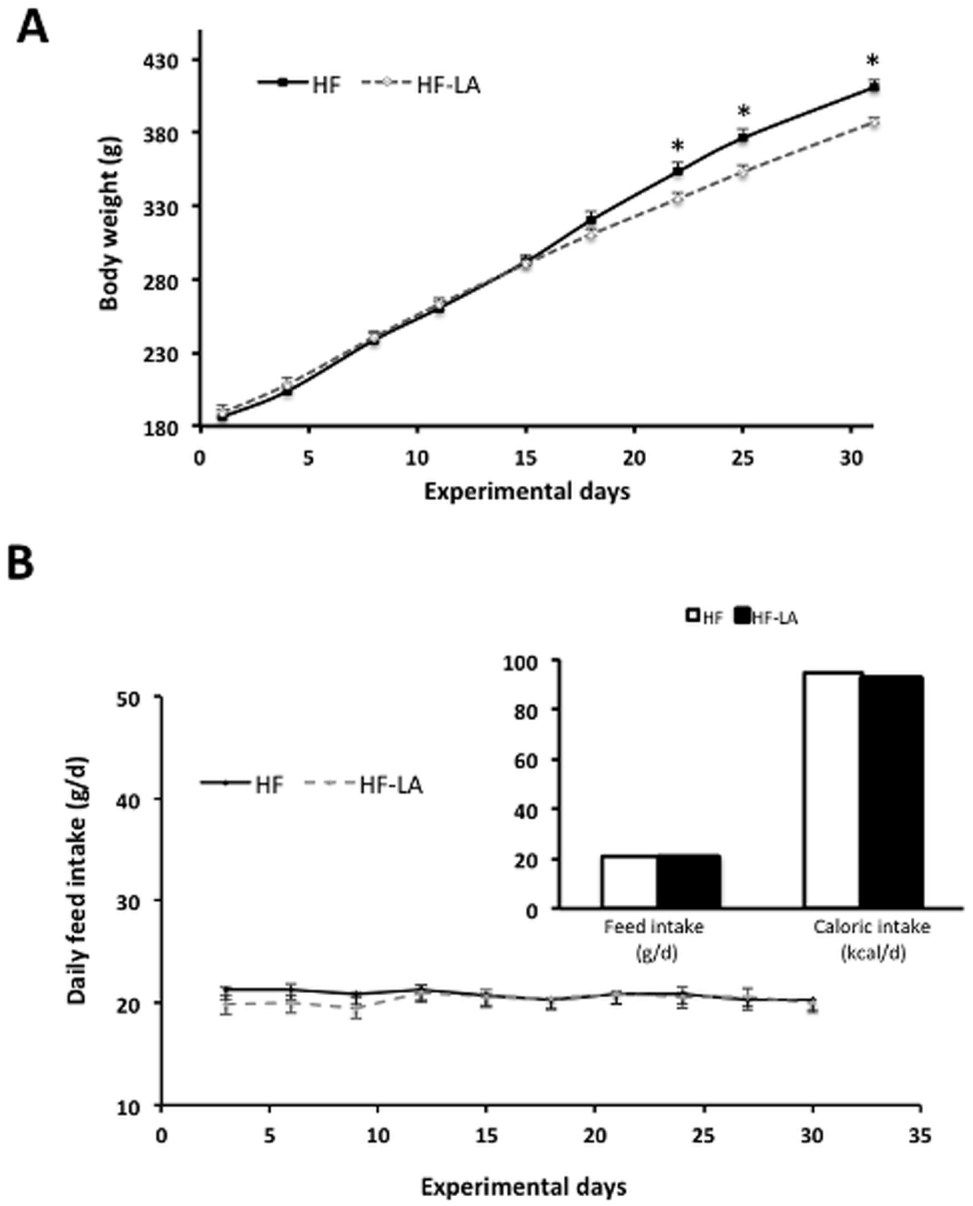
Growth and feed/caloric intake of Zucker rats fed a high fat (HF) diet or the HF diet supplemented with 0.25% α-lipoic acid (HF-LA) for 30 days. (A) Body weight time course over the 30-day experiment; (B) Daily feed intake over the course of the 30-day experiment and average daily feed and total caloric intake (insert). *, denotes a significant difference (p<0.05); n = 8/group.

Consumption of LA protected against body weight gain over the duration of the 30-day experiment compared with the HF group (Fig. 1A). The LA-supplemented animals showed a significantly reduced body weight during the last week of the experimental period (days 22, 25, and 30) compared with the HF group. Although average starting body weight was similar between the two groups (HF, 185.8±4.6 g; HF-LA, 188.6±3.8 g), percent body weight change was significantly reduced (*p*<0.05) in the HF-LA group (102.0±3.1% change) versus animals receiving the HF diet (120.6±3.7% change). As daily feed and total caloric intake for the duration of the 30-day feeding trial did not differ between the two groups (Fig. 1B), the protective effect of LA against diet-induced weight gain was independent of its anorectic properties.

### Plasma lipid and lipoprotein response

**Figure 2:**
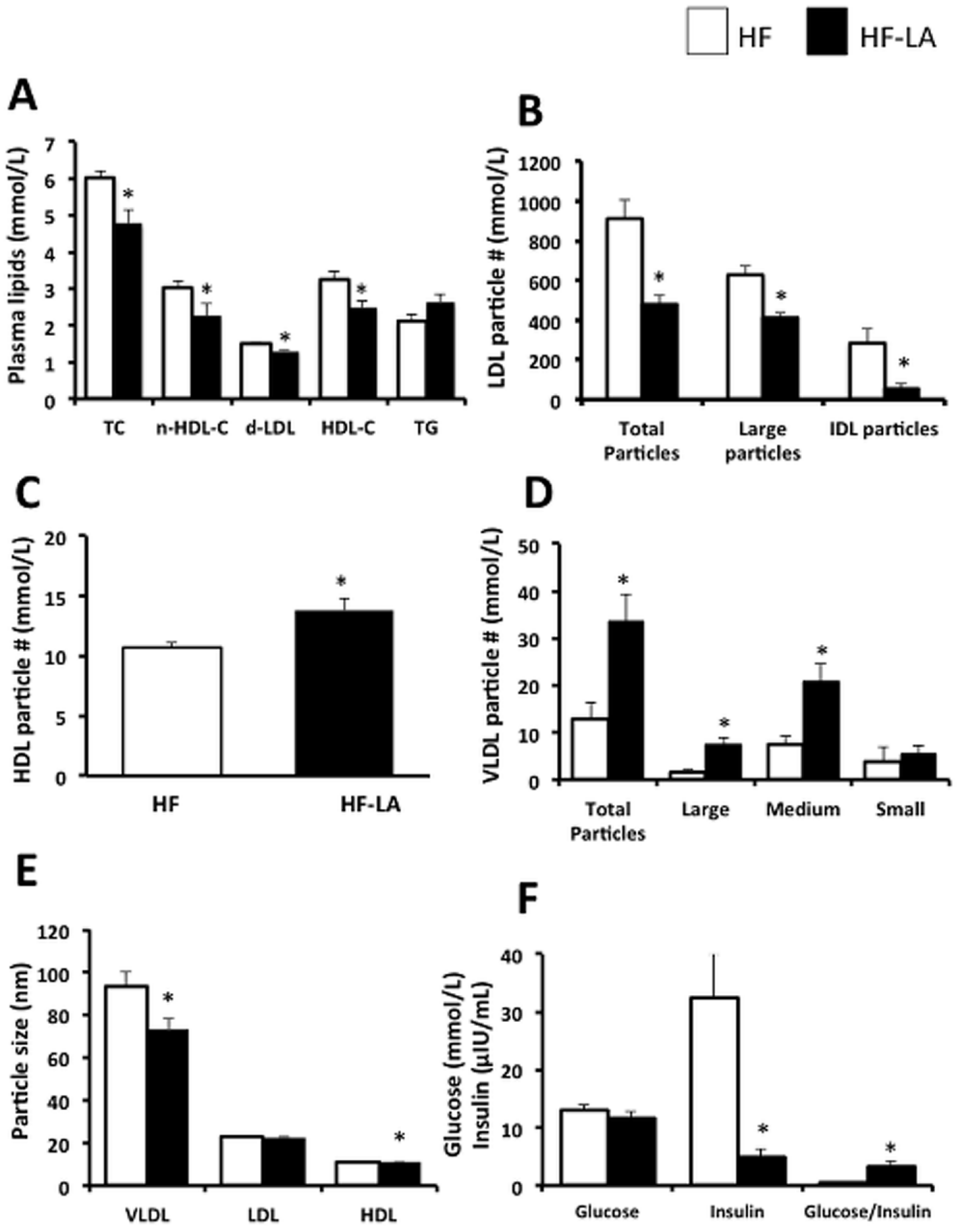
Blood lipid, lipoprotein, glucose and insulin responses in Zucker rats fed a high fat (HF) diet or the HF diet supplemented with 0.25% α-lipoic acid (HF-LA) for 30 days. (A) Plasma lipids including total cholesterol (TC), non-HDL cholesterol (n-HDL-C), direct low density lipoprotein cholesterol (d-LDL-C), high density lipoprotein cholesterol (HDL-C), and triglycerides (TG); (B) LDL particle number; (C) HDL particle number; (D) VLDL particle number; (E) lipoprotein particle size; and (F) glucose and insulin. *, denotes a significant difference (p<0.05); n = 8/group.

Consumption of LA reduced (*p*<0.05) plasma TC (−21%), non-HDL-C (−25%), d-LDL-C (−16%) and HDL-C (−23%) compared with the HF group (Fig. 2A). No difference (*p*>0.05) was observed in plasma TG between the HF and HF-LA groups (Fig. 2A). LA consumption reduced (*p*<0.05) total LDL particle number (−47%), a consequence of reductions in both large (−34%) and intermediate (−81%) LDL particles compared with the HF animals (Fig. 2B). Compared with the HF group, LA-fed animals demonstrated an increase (*p*<0.05) in the number of total HDL particles (22%, Fig. 2C) and total VLDL particles (160%, mainly associated with increases in the large and medium fractions, Fig. 2D). Compared with the HF group, LA consumption reduced (*p*<0.05) the size of VLDL (−21%) and HDL (−5%) particles but did not affect (p>0.05) the size of circulating LDL particles (Fig. 2E). LA supplementation reduced (*p*<0.05) plasma insulin (84%) and increased the glucose/insulin ratio by 6 fold (a marker of insulin sensitivity) without altering (*p*>0.05) plasma glucose concentrations (Fig. 2F).

### Hepatic response

LA-supplementation differentially modulated the relative content of hepatic fatty acids (% total fatty acids) by increasing (*p*<0.05) myristic (14∶0), linoleic (18∶2), and archidonic (20∶4) concentrations and reducing palmitic (16∶0) and palmitoleic (16∶1) concentrations. Total fatty acid concentration (total peak area normalized to the internal standard, heptadecanoic acid) was decreased (37%, p<0.05) in the LA-supplemented animals compared with the HF group (Table 2). The LA group demonstrated a reduction (80%, *p*<0.05) in hepatic TG content compared with the HF group (Table 2). No difference (*p*>0.05) was observed in hepatic cholesterol concentration between the HF and LA supplemented animals (Table 2).

**Table 1:** Formulation of high fat and LA-supplemented diets fed to Zucker rats.

	DIETS	
Ingredient^1^	HF^2^	HF-LA^3^
Sucrose	32.12	32.12
Milk fat	19.96	19.96
Corn oil	3.0	3.0
Casein	19.47	19.47
Maltodextrin	9.98	9.98
Cornstarch	4.99	4.99
Cellulose	4.99	4.74
LA	0	0.25
AIN76-mineral mix	3.49	3.49
AIN76-vitamin mix	0.99	0.99
Calcium carbonate	0.39	0.39
DL-methionine	0.29	0.29
Choline bitrate	0.19	0.19
Cholesterol	0.14	0.14
Ethoxyquin	0.004	0.004
Total	100	100
*Macronutrient Profile (% energy)*		
Protein	15.8	15.8
Carbohydrate	44.2	44.2
Fat	40.0	40.0

1 % composition.

2 HF, high fat diet.

3 HF-LA, high fat diet supplemented with 0.25% α-lipoic acid.

**Table 2:** Hepatic fatty acid composition (% total fatty acids), triglyceride (mmol/g tissue), and cholesterol concentration (mg/g tissue) in Zucker rats fed a high fat (HF) diet or the HF diet supplemented with 0.25% α-lipoic acid (HF-LA) for 30 days.

Variable	HF^1^	HF-LA^2^
**Fatty acid (% total fatty acids)**		
Myristic (14∶0)	3.50±0.22	4.90±0.21*
Myristoleate (14∶1)	0.63±0.04	0.63±0.04
Palmitic (16∶0)	38.97±0.54	31.15±0.53*
Palmitoleic (16∶1)	12.19±0.66	8.5±0.28*
Stearic (18∶0)	2.77±0.11	3.18±0.64
Oleic (18∶1)	35.29±1.12	34.20±0.56
Linoleic (18∶2)	5.58±0.61	14.44±0.65*
Arachidonate (20∶4)	1.03±0.13	2.96±0.14*
**Total fatty acids (total peak area)** 3	194.03±20.58	121.99±10.77*
**Triglycerides (mmol/g tissue)**	2.21±0.30	1.22±0.30*
**Cholesterol (mg/g tissue)**	6.06±1.06	7.52±1.33

1 HF, high fat diet.

2 HF-LA, high fat diet supplemented with 0.25% α-lipoic acid.

3 normalized to internal standard, heptadecanoic acid.

*p<0.05; values are mean ± SE (n = 8/group).

**Figure 3:**
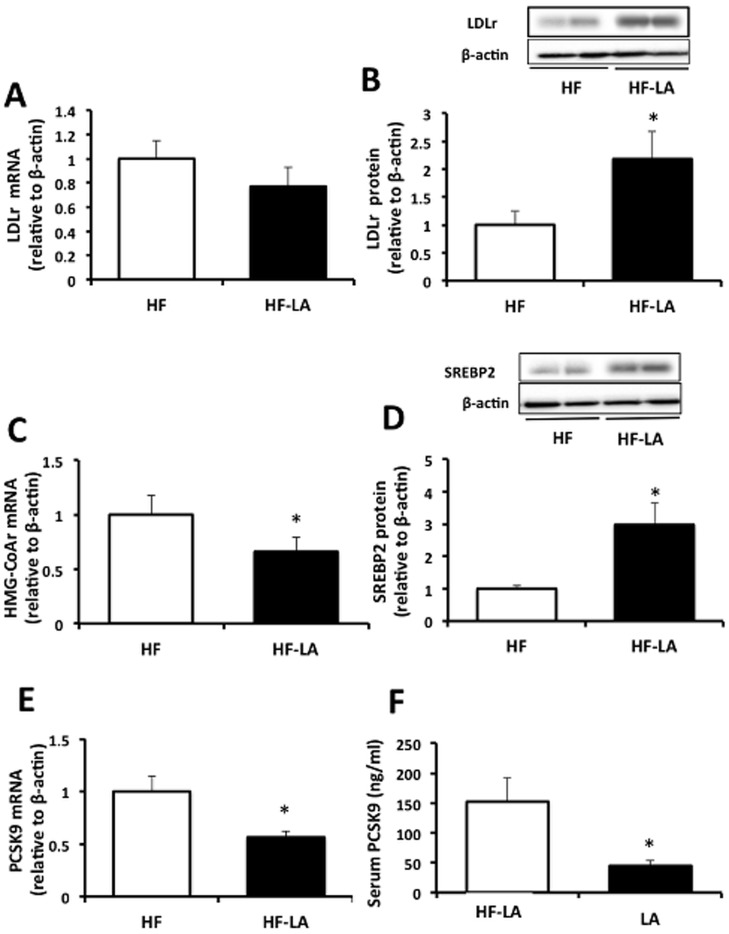
Hepatic expression of cholesterol responsive genes and blood PCSK9 in Zucker rats fed a high fat (HF) diet or the HF diet supplemented with 0.25% α-lipoic acid (HF-LA) for 30 days. (A) low density lipoprotein receptor (LDLr) mRNA; (B) (B) low density lipoprotein receptor protein abundance; (C) 3-hydroxy-3-methyl-glutaryl-CoA reductase mRNA (HMG-CoAr); (D) Nuclear sterol regulatory element binding protein abundance (SREBP2); (E) Proprotein convertase subtilisin/kexin type 9 mRNA (PCSK9); and (F) Serum PCSK9 concentrations (ng/mL). *, denotes a significant difference (p<0.05). All data normalized to β-actin and expressed relative to HF group; n = 8/group.

Although no change in LDLr mRNA was observed between the two groups (Fig. 3A, the LA-supplemented animals demonstrated enhanced (*p*<0.05) protein abundance of LDLr (2 fold of HF, Fig. 3B). LA-supplementation also resulted in a reduction (*p*<0.05) in the mRNA expression of HMG-CoAr (0.7 fold of HF, Fig. 3C) and an increase in nuclear SREBP2 abundance (3 fold of HF, Fig. 3D). LA supplementation was also associated with a reduction in hepatic PCSK9 mRNA (0.5 fold of HF, Fig. 3E) and in serum concentration PCSK9 (70%, Fig. 3F), a primary regulator of LDLr turnover.

**Figure 4:**
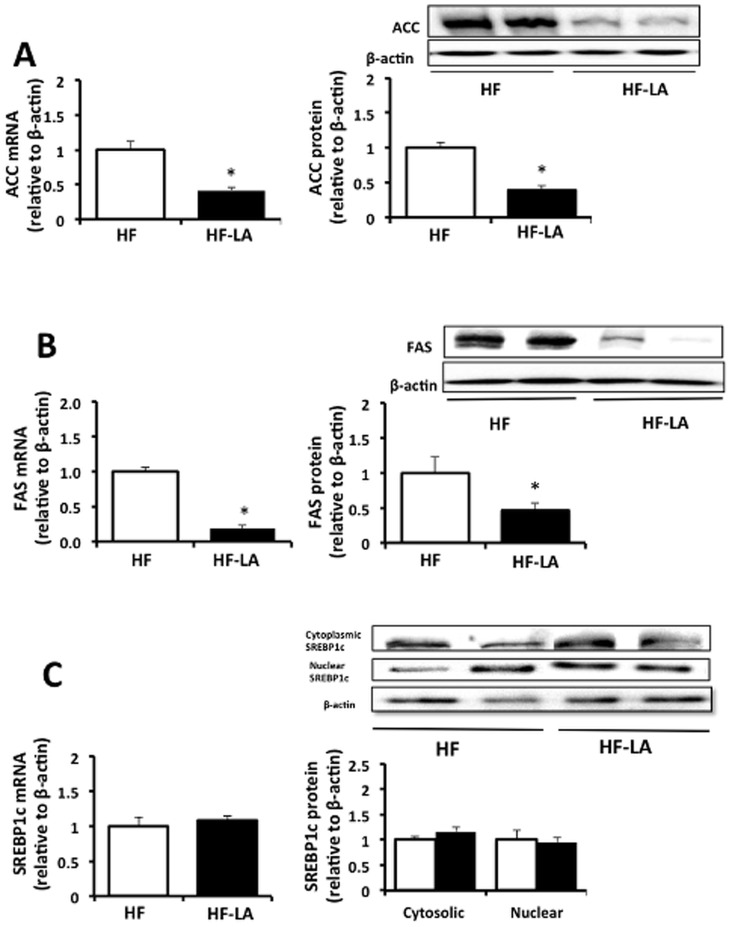
Hepatic expression of lipogenic regulators in Zucker rats fed a high fat (HF) diet or the HF diet supplemented with 0.25% α-lipoic acid (HF-LA) for 30 days. (A) Acetyl-CoA carboxylase (ACC) mRNA expression and protein abundance; (B) Fatty acid synthase mRNA expression and protein abundance (FAS); (C) cytoplasmic and nuclear sterol regulatory element binding protein 1c (SREBP1C) mRNA and protein. *, denotes a significant difference (p<0.05). All data normalized to β-actin and expressed relative to HF group; n = 8/group.

Compared with the HF group, expression of hepatic lipogenic targets was reduced (*p*<0.05) in the LA group, including ACC mRNA and protein (0.4 and 0.5 fold of HF, respectively) and FAS mRNA and protein (0.18 and 0.46 of HF, respectively) (Fig. 4A, B). The mRNA expression and protein abundance of SREBP1c, a major transcriptional regulator of lipogenesis, did not differ (*p*>0.05) between the LA and HF groups (Fig. 4 C). To examine the direct effects of LA on hepatic gene and protein expression, a normal rat hepatocyte cell line was treated with various doses of LA. We detected no difference (*p*>0.05) in the mRNA expression of ACC or FAS in rat hepatocytes exposed to LA (50–300 µM) compared with controls (Fig. S1A). Cells exposed to 600 µM LA demonstrated a reduction (p<0.05) in the protein abundance of ACC (−3.1 fold of control, Fig. S1B) and a tendency (p = 0.10) for reduced FAS abundance compared with the control group (−1.4 fold of control, Fig. S1C).

**Figure 5:**
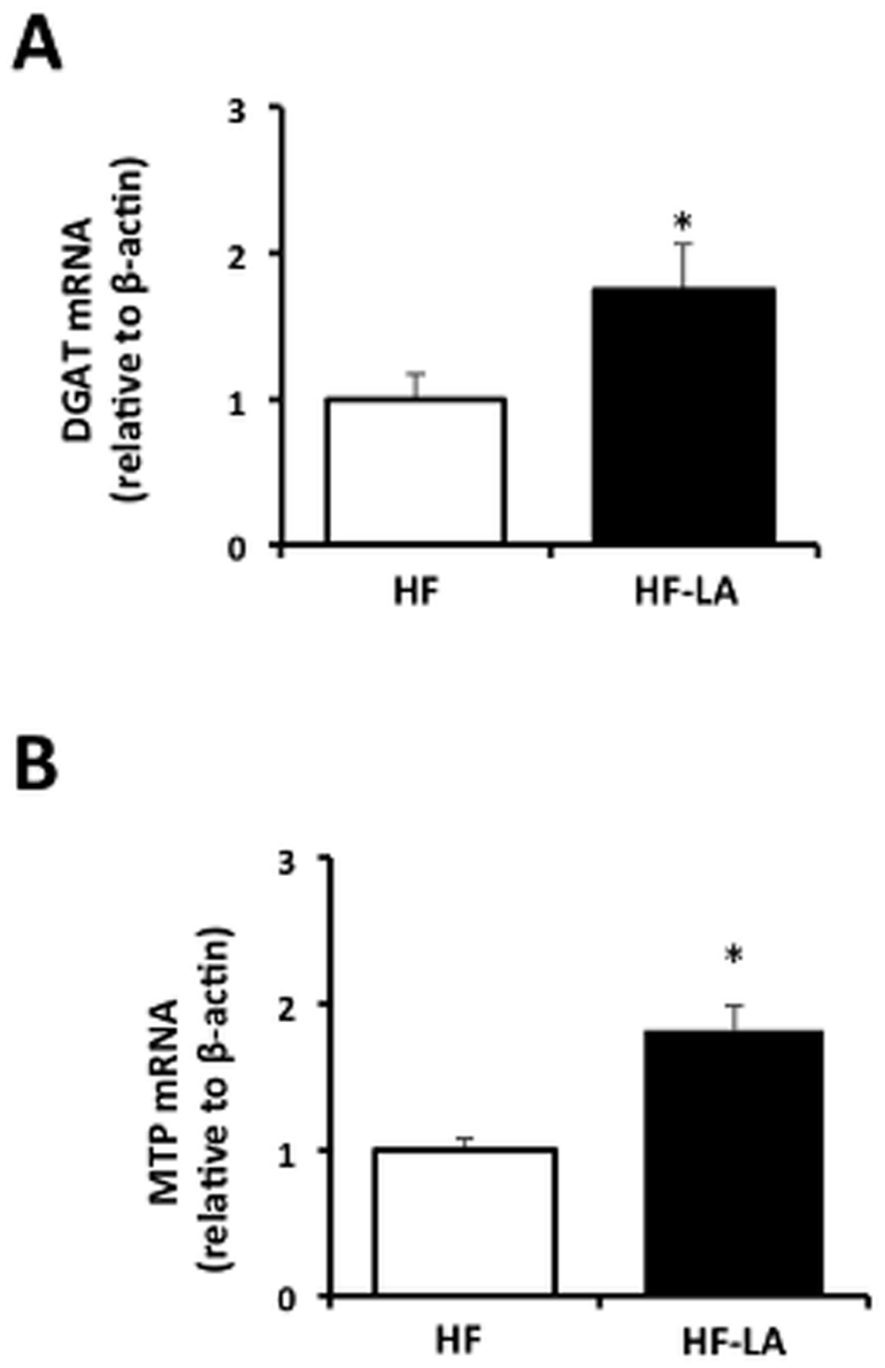
Targets of hepatic triglyceride synthesis/VLDL production regulators in Zucker rats fed a high fat (HF) diet or the HF diet supplemented with 0.25% α-lipoic acid (HF-LA) for 30 days. Diacylglycerol acyltransferase (DGAT) mRNA expression; (D) Microsomal triglyceride transfer protein (MTP) mRNA expression; n = 8/group.

Hepatic targets involved in TG synthesis and VLDL packaging were elevated (*p*<0.05) in response to LA feeding compared with the HF group, including DGAT (1.8 fold of HF) and MTP mRNA (1.8 fold of HF) (Fig. 5A, C).

**Figure 6:**
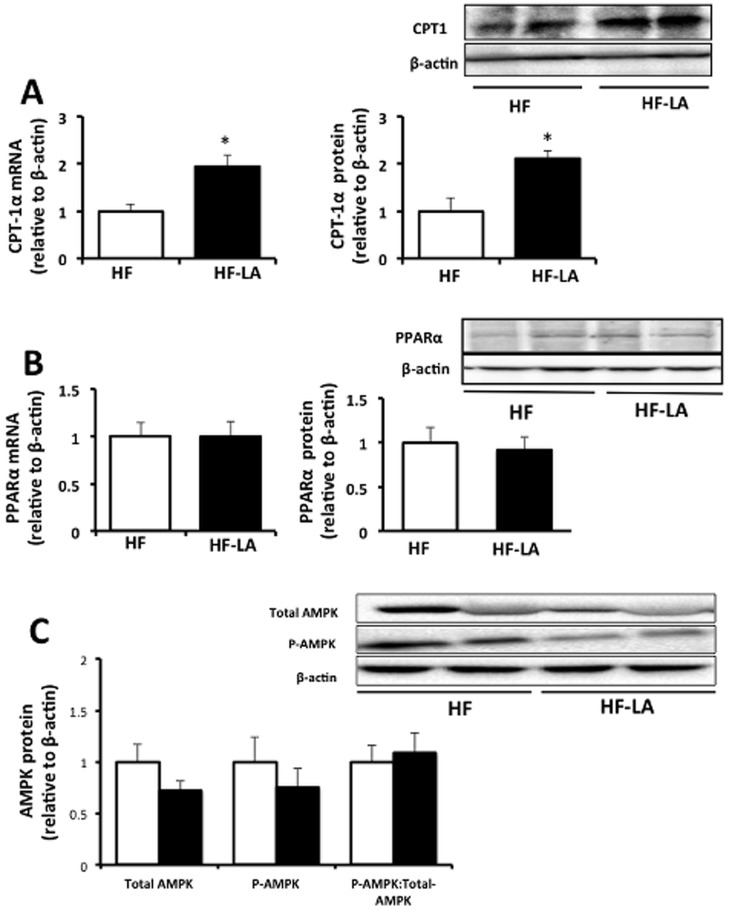
Expression of hepatic fat oxidative targets in Zucker rats fed a high fat (HF) diet or the HF diet supplemented with 0.25% α-lipoic acid (HF-LA) for 30 days. (A) Carnitine palmitoyltransferase I (CPT1α) mRNA expression and protein abundance; (B) PPARα mRNA expression and protein abundance; and (C) P-AMPK, total AMPK, and total:P-AMPK. *, denotes a significant difference (p<0.05). All data normalized to β-actin and expressed relative to HF group; n = 8/group.

Examination of hepatic fat oxidative targets revealed an increase (*p*<0.05) in the mRNA and protein expression of CPT1α in the LA animals compared with the HF group (mRNA, 1.9 fold of HF; protein, 2.1 fold of HF, Fig. 6A). However, no difference (*p*>0.05) was observed in PPARα or total AMPK or P-AMPK, key regulators of hepatic fatty acid oxidation, between the LA and HF groups (Fig. 6B,C).

### Muscle response

**Figure 7:**
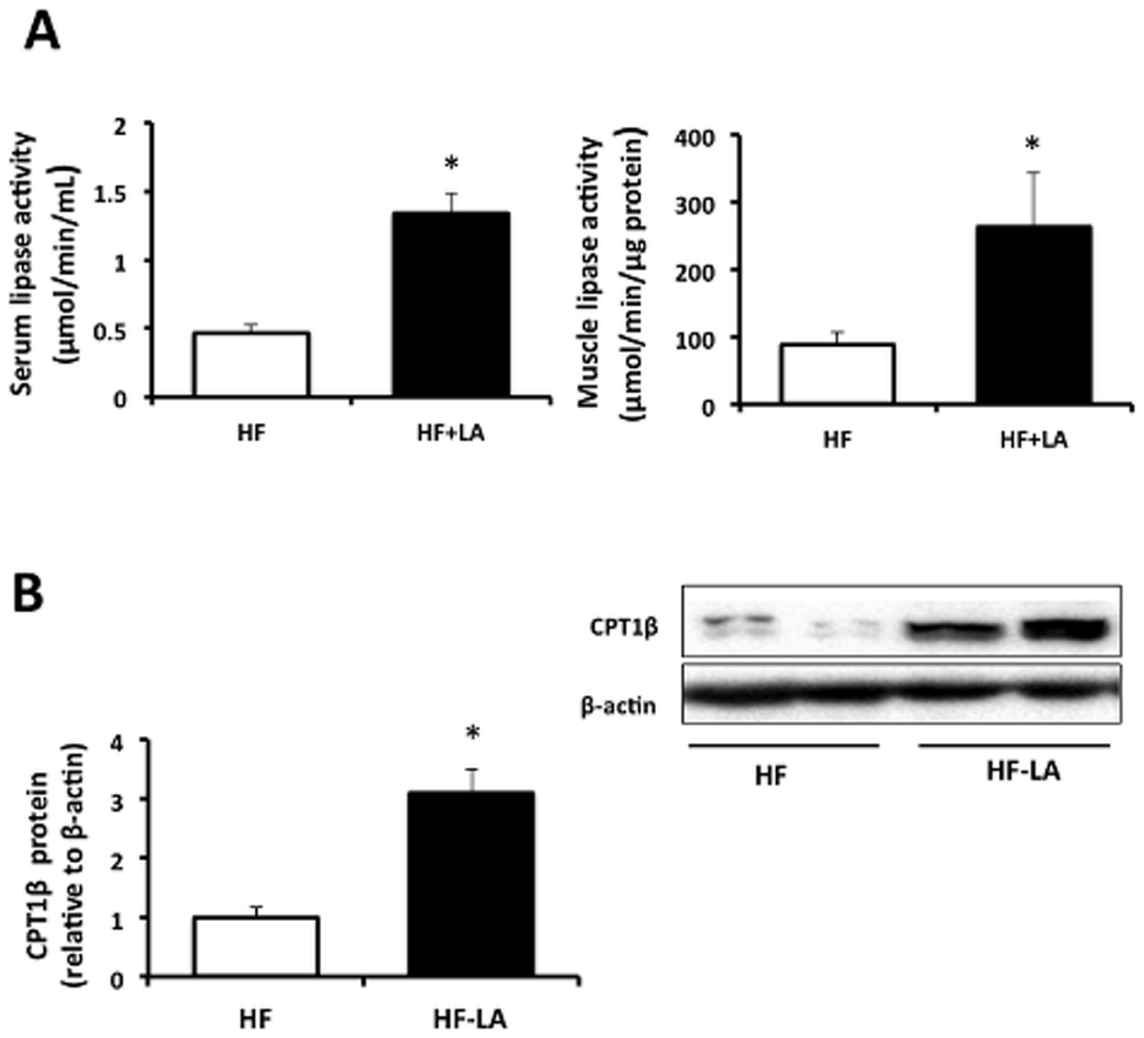
Expression of skeletal muscle fatty acid uptake and oxidation markers in Zucker rats fed a high fat (HF) diet or the HF diet supplemented with 0.25% α-lipoic acid (HF-LA) for 30 days. (A) Total lipase activity in serum and muscle total protein tissue extracts (n = 6 HF; 5 LA); (B) carnitine palmitoyltransferase I (CPT1β). *, denotes a significant difference (p<0.05). All data normalized to β-actin and expressed relative to HF group; n = 8/group.

LA-supplemented animals demonstrated higher (p<0.05) total lipase activity in both serum (2.9 fold, Fig 7A) and muscle total tissue extracts (2.8 fold, Fig. 7A) compared with the HF group. CPT1β protein abundance was increased (p<0.05, 3 fold of HF, Fig. 7B) in the LA-supplemented rats compared with the HF group.

## Discussion

Using a Zucker rat model driven by both a genetic predisposition and diet induction toward obesity and dyslipidemia, results of this study expands the current knowledge base regarding the protective properties of LA with several novel observations. First, in addition to the previously reported reductions in serum TC (−21%), non-HDL-C (−25%), and LDL-C (−16%), LA supplementation reduced LDL particle number (−47%) and increased HDL particle number (22%) compared with the HF-fed animals. This hypocholesterolemic response was associated with a reduction in hepatic PCSK9 mRNA (−0.5 fold) and serum PCSK9 concentrations (−70%) and a related increase in hepatic LDLr protein abundance (2 fold). Second, in spite of excessive fat intake and a predisposition to fatty liver, LA supplementation protected against hepatic TG accumulation (−80%), likely through multiple mechanisms including reduced *de novo* lipogenesis, enhanced VLDL export, and increased fat oxidation in both the liver and muscle.

The reductions in blood TC and non-HDL-C cholesterol in the LA-supplemented animals has been reported in multiple previous pre-clinical studies [18]–[21], but not all [15], [22]. This cholesterol-lowering response may be related to the anti-obesity effects of LA observed in this study and previous animal work [23], [24], but less consistently in human LA interventions [9], [25], [26]. We are not aware of previous work that has examined LA supplementation on lipoprotein distribution patterns; however, the reduction in LDL-particle number in the LA-supplemented rats may have important implications given the strong correlation between LDL particle size and CVD risk [27]. Although HDL-C concentrations were reduced in response to LA, the atherogenic consequences of this are unclear as we also detected an increase in HDL particle number following LA supplementation. Mackey et al. (2012) recently reported that, unlike HDL-C, HDL-particle number is independently associated with carotid intima-media thickness and incident coronary events after adjusting for LDL-particle number [28].

Studies examining the molecular mechanisms underlying the cholesterol-lowering properties of LA are limited. The observed modulation in blood cholesterol concentrations and lipoprotein distribution may be related to a reduction in hepatic cholesterol synthesis and enhanced cholesterol clearance, as demonstrated by a reduction in the mRNA expression of HMG-CoAr (Fig. 3C), the rate-limiting enzyme in cholesterol synthesis, and enhanced abundance of LDLr protein (Fig. 3B). Our data further suggests that enhanced hepatic LDLr protein abundance may be mediated through a reduction in serum PCSK9, a mechanism not previously investigated. Current research is heavily focused on pharmacological inhibition of blood PCSK9 concentrations with RNA interference or antibody-based therapies [29]–[32], though research into diet and nutraceutical options to modulate PCSK9 activity has received less attention [33]–[35]. Although the transcriptional regulation of hepatic LDLr expression is known to be mediated through SREBP2 [36], turnover of the LDLr protein is regulated by PCSK9, a serine endoprotease that promotes degradation of the LDLr protein [37]. Previous work suggests that PCKS9 is predominately synthesized and secreted by the liver to initiate extracellular degradation of membrane-incorporated LDLr following direct binding [38]. As serum PCSK9 concentration is reflective of LDLr activity and considered a potentially important biomarker of cardiovascular disease risk [39], results of this study warrant further investigation in both animal and human investigations.

To account for changes observed in the expression of intracellular cholesterol regulatory genes, we assessed the nuclear abundance of SREBP2, the master transcriptional regulator of cholesterol responsive genes. The observed increase in nuclear SREBP2 protein is difficult to reconcile given the absence of a change in intracellular cholesterol concentrations and no corresponding increase in SREBP2 target gene mRNA expression including PCSK9, HMG-CoAr and LDLr. In fact, hepatic HMG-CoAr mRNA was reduced in the LA-supplemented animals (Fig. 3C), suggesting a possible reduction in hepatic cholesterol synthesis. As HMG-CoAr transcription has been shown to be increased by insulin [40], it is possible that the large reduction in insulin observed with LA-supplementation may have contributed to a lower HMG-CoAr mRNA levels compared with the HF group, even in the face of increased SREBP2 abundance.

Through a reduction in hepatic TG and total fatty acids (Table 1), livers of LA supplemented animals were protected against diet-induced fat accumulation, likely through multiple mechanisms. First, hepatic mRNA and protein expression patterns suggest that LA supplementation led to an inhibition of *de novo* fat synthesis (ACC and FAS) and increased fatty acid oxidation (CPT1α). We explored several potential mechanisms to account for the apparent shift in hepatic fat metabolism from synthesis to oxidation in the LA group. Our *in vitro* data suggests that the reduced lipogenic response may not be a direct effect of LA as hepatocytes exposed to LA at 300 µM did not demonstrate significant reductions in ACC or FAS mRNA or protein abundance. However, cells exposed to a supraphysiologial dose of 600 µM of LA did demonstrate a reduction in ACC abundance and a tendency for reduced FAS abundance (Fig. S1). This reduction in ACC and FAS expression in the LA-supplemented animals does also not appear to be related to changes in nuclear SREBP1c abundance, the master-transcriptional regulator of fatty acid synthesis (Fig. 4C). Although a reduction in the insulin-induced stimulation of SREBP1c is an attractive hypothesis to account for the reduction in lipogenic gene expression in response to LA, the literature on this matter seems inconclusive. Park et al. (2008) demonstrated a reduction in hepatic SREBP1c protein expression in HF-fed rats supplemented with LA [16]. However, Butler et al. (2009) reported a reduction in precursor SREBP1c in total liver extracts but no change in nuclear SREBP1c abundance in LA-supplemented Zucker diabetic rats[22]. Similar to our results, Huang et al. (2007) reported a reduction in plasma insulin but no corresponding decrease in SREBP1c mRNA expression in Sprague Dawley rats fed ad-libitum with diets containing 1, 2.5, and 5 g LA/kg [15]. The lack of change in SREBP1c in the current study may be associated with the fasting state (14 h) that the animals were in at the time of tissue collection. It is possible that potential LA-induced inductions of SREBP1c may be more readily detectible in the fed state when lipogenesis is maximally stimulated.

Alternatively, changes in the hepatic fatty acid profile in the LA rats may have contributed to the reduced lipogenic response. LA supplementation increased the proportion of the polyunsaturated fatty acids linoleic (18∶2) and arachidonic (20∶4), both of which have been shown to be potent inhibitors of hepatocyte lipogenic gene expression [41], [42]. Finally, we did not detect changes in the hepatic protein abundance of PPARα or total and phosphorylated AMPK, key intracellular regulators of fat oxidation [43]. Although LA has been shown to stimulate AMPK activity in both the liver [16] and muscle [44], this stimulation appears to manifest as a temporary response to acute intraperitoneal LA injection (∼24 h) or short term feeding scenarios (3–4 days) that is not sustained in the longer-term [16], [45].

The substantial increase in VLDL (160%) contrasts with the purported TG lowering effects of LA reported previously [22], [24], [46], [47], however, the vast majority of these studies have fed chow or low-fat diets [22], [24], [47] compared with the HF-diet (40% of energy) used in our study. This increase in VLDL particles could be due to multiple factors including modulation of hepatic VLDL synthesis/secretion or reduced peripheral remodeling of VLDL to LDL. The enhanced mRNA expression of hepatic DGAT1 and MTP, two enzymes that are associated with increased secretion of TG-rich apoB–containing lipoproteins [48], [49], is supportive of a potential increase in VLDL secretion. It is possible that diminished lipoprotein lipase activity could have contributed to an increase in VLDL particles and resulted in the substantial reduction in LDL particles observed in the LA supplemented group. However, as serum and muscle total lipase activity was enhanced upon LA-supplementation, our data does not support an impairment of VLDL to LDL conversion but rather an increase in peripheral fatty acid uptake. Previous work has shown that serum LPL circulates with lipoproteins to promote tissue binding and is positively associated with VLDL triglyceride [50], [51]. We further observed an increase in muscle CPT1β abundance in the LA versus the HF group. Overall, the totality of the data support the hypothesis that LA-supplementation protected against hepatic fat accumulation by enhancing VLDL export for peripheral uptake and oxidation. In a similar fashion, nopal, a catus-derived functional food with lipid-lowering and antioxidant properties, was recently shown to attenuate hepatic steatosis by enhancing VLDL export in Zucker rats [52]. To further elucidate the potential protective effects of LA on TG metabolism when consuming excessive dietary fat, further studies that directly examine lipoprotein kinetics are required.

Much of the modulation of hepatic fat metabolism in the LA-supplemented animals, particularly with respect to *de novo* synthesis and VLDL production, may be associated with the observed reduction in plasma insulin and an improvement in the glucose/insulin ratio, an effect that has been seen in previous studies [24], [53]. Insulin exerts substantial influence on hepatic lipid metabolism by stimulating hepatic lipogenesis through SREBP1c [54] and suppressing VLDL-TG secretion by reducing apoB100 synthesis [54], [55]. It is possible that the dramatic reduction in fasting insulin (84%) that we observed in the LA-supplemented group may have blunted the repressive effects of insulin on hepatic VLDL-TG secretion and caused an increase in plasma VLDL particle concentration in comparison with the HF group.

In summary, the current work demonstrates the protective effects of LA against hypercholesterolemia and hepatic fat accumulation under conditions of strong genetic and dietary predisposition toward obesity and dyslipidemia. Study results indicate that, independent of changes in feed and caloric intake, LA-supplementation reduces plasma cholesterol likely through a PCSK9-dependent mechanism and protects against hepatic fat accumulation through multiple mechanisms that may involve a shift in fat metabolism toward oxidation and enhanced VLDL export for peripheral oxidation.

## Methods

### Animals and diets

Sixteen 160 g male Zucker rats (Harlan Laboratories, Indianapolis, Indiana) were brought to the Animal Care Facility at the University at Buffalo. Rats were housed individually in cages with shavings in a temperature-controlled room (20°C) with a 12 h light/dark cycle and had free access to water. At the initiation of the experiment, rats were randomly assigned to 1 of 2 diets (n = 8/group) for 30 days according to Table 1 :(i) high fat diet (HF, AIN 76A Western Diet, 40% energy from fat) or (ii) HF diet supplemented with 0.25% LA (HF-ALA, R enantiomer, ALA R+ SAP, Nutritional Fundamentals for Health, Quebec, Canada). Diet ingredients (Dyets, Bethlehem, PA) were mixed on site in two separate batches and stored at 4°C for the duration of the experiment. Rats were fed daily and any leftover feed was discarded. Dietary spillage was included in daily feed intake estimates. As LA possesses anorectic properties [16], [17], all animals were pair-fed to ensure similar feed and caloric intake between the HF and HF-LA groups. Body weights were obtained 3 times per week. The animals used in this experiment were cared for in accordance with the guidelines established by the Institutional Animal Care and Use Committee (IACUC). All procedures were reviewed and approved by the Animal Care Committee at the University at Buffalo (protocol # PTE25061N).

### Sample collection and processing

Following the 30 day feeding period, rats were anesthetized with isoflurane for blood and tissue collection. Fasting (14vh) blood (serum and EDTA plasma) was collected by cardiac puncture and processed as previously described [56] and stored at −80°C. The liver and plantar flexor was quickly excised, rinsed in chilled saline (pH 7.4, 154 mM containing 0.1 mM phenylmethylsulfonyl fluoride) and flash frozen in liquid nitrogen. All tissues were stored at −80°C until further processing and analyses.

### Blood biochemistry

Plasma total cholesterol (TC), high-density lipoprotein cholesterol (HDL-C), non-HDL cholesterol (n-HDL-C), and TG were determined by automated enzymatic kits (Sekisui Diagnostics, Lexington, MA, USA) on an ABX Pentra 400 autoanalyzer (Horiba Instruments Inc., Irvine CA, USA). Direct low-density lipoprotein cholesterol (d-LDL) was assessed with a commercial Elisa kit (KT-60293, Kamiya BioMedical Company, Seattle, WA, USA). Direct assessment of lipoprotein particle number and size was conducted by nuclear magnetic resonance spectroscopy (NMR) using automated signal acquisition followed by computational analysis and proprietary signal processing algorithms (Liposcience, Raleigh, NC) [57]. Serum insulin was analysed by ELISA (EZRMI-13K, Millipore, Billerica, MA) and glucose was measured by colorimetric analysis (ab6533, abcam, Cambridge, MA). Proprotein convertase subtilisin/kexin type 9 (PCSK9) was measured in serum samples using a commercial ELISA kit (CY-8078, MBL International, Woburn, MA).

### Hepatic lipids

Hepatic TG were analyzed with a commercial kit (ab65336, Abcam, Cambridge, MA, USA). Hepatic TG were extracted by homogenization in aqueous Triton-X buffer (2%) and measured at OD_570 nm_ according to manufacturer instructions. For analysis of hepatic fatty acids, approximately 0·5 g of pulverized liver was spiked with heptadecanoic acid (C17:0) as internal standard. Total lipids were isolated from liver tissue with a modified Dole mixture (3 hepatane:12 propanol:3 DDH_2_O, vol:vol) followed by extraction with heptane: DDH_2_O (3∶1 vol:vol) [58]. Fatty acid extracts were methylated with methanolic boron trifluoride (Sigma Aldrich, St. Louis, MO). Fatty acid methyl esters were separated using a Supelcowax 10 column (30 m×0·25 mm with 0·25 m film thickness; Supelco, Bellefonte, PA, USA) in a Shimadzu GC-17A gas chromatograph fitted with a flame ionization detector. Relative hepatic fatty acid content was calculated by using individual FA peak area relative to the total area and expressed as the percentage of total fatty acids.

Hepatic cholesterol was extracted and analyzed according to our previously published procedures [56], [59]. Approximately 500 mg of pulverized liver was spiked with α-cholestane as internal standard and saponified in freshly prepared KOH–methanol at 100°C for 1 h. The non-saponifiable sterol fraction was extracted with petroleum diethyl ether and dried under N_2_ gas. Sterol fractions were analyzed on the same GC system using a SAC-5 capillary column (30 m×0·25 mm×0·25 mm, Supelco, Bellefonte, CA, USA).

### Lipase Activity

Total lipase activity in serum and total muscle tissue extracts was analyzed using a commercial lipase activity assay kit (Cayman Chemical, Ann Arbor, MI, USA) according to manufacturer's instructions.

### RNA preparation and real-time RT-PCR

Total RNA was isolated from whole liver tissue using TRIzol reagent (Invitrogen Inc., Grand Island, NY). RNA concentration and integrity was determined with spectrophotometry (260 nm) and agarose gel electrophoresis, respectively. RNA preparation and real-time RT-PCR was conducted using a one-step QuantiFast SYBR Green RT-PCR kit (Qiagen Inc., Valencia, CA) on a Biorad MyiQ real time PCR system according to previously established protocols [60]. Gene expression was analyzed using the 2(-delta delta Ct) method [61]. Sequences of sense and antisense primers for target and housekeeping genes were based on previously published reports for β-actin [62], Low-density lipoprotein receptor (LDLr) [63], sterol-regulatory element-binding protein 1c (SREBP1c) [64], peroxisome proliferator-activated receptor alpha (PPAR) [65], acetyl-coA carboxylase (ACC) [66], fatty acid synthase (FAS) [67], acyl-coA oxidase 1 (ACOX) [66], diacylglycerol acyltransferase (DGAT) [66], microsomal triglyceride transfer protein (MTP) [66], and carnitine palmitoyltransferase 1α (CPT1α) [62], 3-hydroxy-3-methylglutaryl-coenzyme A reductase (HMG-COAr) [68], proprotein convertase subtilisin/kexin type 9 (PCSK9) [69]; and sterol regulatory element-binding protein 2 (SREBP2) [70].

### Immunoblot analysis

Immunoblots were prepared as previously described [60]. Tissue was homogenized in 10 volumes of CHAPS-containing buffer [40 mM HEPES (pH 7.5), 120 mM NaCl, 1 mM EDTA, 10 mM pyrophosphate, 10 mM -glycerophosphate, 40 mM NaF, 1.5 mM sodium vanadate, 0.3% CHAPS, 0.1 mM PMSF, 1 mM benzamidine, 1 mM DTT, and Roche complete protease inhibitors (#04693116001, Roche, Indianapolis, IN)]. The resulting homogenate was clarified by a 1,000×*g* for 5 minutes (at 4°C), and the supernatant was retained (i.e. cytoplasmic fraction). The remaining pellet was washed with CHAPS buffer three times, followed by a 1,000×*g* centrifugation for 5 minutes (at 4°C), then resuspended in 60 µl of lysis buffer, and 8.3 µl of 5 M NaCl was added to lyse the nuclei. This mixture was rotated at 4°C for 1 hour and then centrifuged at 12,000×*g* for 15 minutes (at 4°C). The subsequent supernatant contained the soluble nuclear-enriched fraction. A small aliquot of each fraction was taken for the determination of protein concentration for each sample. Then, equal volume of 2X sodium dodecyl sulfate loading buffer was added to each fraction for Western analysis. Samples were boiled for 5 minutes, then Western analysis was performed. Cytoplasmic extracts were probed with commercial antibodies specific for 5′ AMP-activated protein kinase (total AMPK, 2531s, Cell Signaling) and phosphorylated AMPK (P-AMPK, 5832s (T172), Cell Signaling), FAS (C2OG5, Cell Signaling), ACC (C83B10, Cell Signaling) and SREBP1c (ab3259, abcam). Nuclear extracts were probed with commercial antibodies for sterol regulatory element binding protein 2 (SREBP2, ab30682, abcam), SREBP1c, and PPARα (ab24509, abcam). Mitochondrial-enriched extracts for CPT1α and β (8F6AE9, abcam; LS-c12435, LifeSpan BioScience) protein abundance were extracted using a commercial kit (Thermo Scientific, Waltham, MA). Target proteins were normalized to β-actin and quantified using Image lab (version 4.1, Biorad Laboratories, Hercules, CA).

### Cell culture

Normal rat liver cells, Clone 9 (ATCC; CRL1493), were maintained and cultured in F-12K (ATCC; 30-2004) medium supplemented with 10% fetal bovine serum, and were incubated in a HERACELL 150i incubator (Thermo Scientific) at 37° celsius and 5% CO_2_. The cells were plated onto 10 cm plates for the RNA isolation experiments at a density of 200,000 cells/well. For the protein expression, the cells were plated at a density of 50,000 cells/well in a 6-well format. The next day, the cells were treated with either 50, 150, 300 µM LA (dissolved in distilled water) or an equal volume of distilled water (control) for 3 h, then harvested in Trizol (Invitrogen) for RNA isolation per the manufacturer's instructions. For protein expression analysis, cells were treated with 300 and 600 µM LA for 3 h then harvested in 1X bromophenol blue dye-free 1X SDS buffer (containing 250 mM tris hydrochloric acid, 500 mM beta-mercaptoethanol, 2% sodium dodecylsulfate (SDS) and 10% glycerol).

### Statistical analyses

Responses between LA and HF rats were compared using an independent t-test [71]. Data were analyzed with SPSS 16 for Mac (SPSS Inc, Chicago IL). Data are presented as mean ± SEM. All results are the means from 8 animals. mRNA expression and protein abundance following hepatocyte exposure to increasing LA dosages were analyzed with a Tukey post-hoc test. Differences were considered significant at *p*≤0.05.

## Supporting Information

Figure S1 Expression of lipogenic targets in rat hepatocytes exposed to α-lipoic acid (LA). (A) mRNA expression of acetyl-CoA carboxylase (ACC) and fatty acid synthase (FAS) exposed to LA for 3 hours at doses of 0 (Con), 50, 150, and 300 µM. (B) Protein abundance of ACC exposed to LA for 3 hours at a dose of 0 (Con), 300 µM and 600 µM. (C) Protein abundance of FAS exposed to LA for 3 hours at a dose of 0 (Con), 300 µM and 600 µM. *, denotes a significant difference (p<0.05) from Con; •, denotes a trend (p = 0.1) from Con. All data normalized to β-actin and expressed relative to HF group.
(TIF)
